# CryoETGAN: Cryo-Electron Tomography Image Synthesis *via* Unpaired Image Translation

**DOI:** 10.3389/fphys.2022.760404

**Published:** 2022-03-04

**Authors:** Xindi Wu, Chengkun Li, Xiangrui Zeng, Haocheng Wei, Hong-Wen Deng, Jing Zhang, Min Xu

**Affiliations:** ^1^Computational Biology Department, Carnegie Mellon University, Pittsburgh, PA, United States; ^2^École Polytechnique Fédérale de Lausanne, Lausanne, Switzerland; ^3^Department of Electrical & Computer Engineering, University of Toronto, Toronto, ON, Canada; ^4^Center for Biomedical Informatics & Genomics, Tulane University, New Orleans, LA, United States; ^5^Department of Computer Science, University of California, Irvine, Irvine, CA, United States

**Keywords:** Cryo-ET, image synthesis, image translation, generative model, generative adversarial network

## Abstract

Cryo-electron tomography (Cryo-ET) has been regarded as a revolution in structural biology and can reveal molecular sociology. Its unprecedented quality enables it to visualize cellular organelles and macromolecular complexes at nanometer resolution with native conformations. Motivated by developments in nanotechnology and machine learning, establishing machine learning approaches such as classification, detection and averaging for Cryo-ET image analysis has inspired broad interest. Yet, deep learning-based methods for biomedical imaging typically require large labeled datasets for good results, which can be a great challenge due to the expense of obtaining and labeling training data. To deal with this problem, we propose a generative model to simulate Cryo-ET images efficiently and reliably: CryoETGAN. This cycle-consistent and Wasserstein generative adversarial network (GAN) is able to generate images with an appearance similar to the original experimental data. Quantitative and visual grading results on generated images are provided to show that the results of our proposed method achieve better performance compared to the previous state-of-the-art simulation methods. Moreover, CryoETGAN is stable to train and capable of generating plausibly diverse image samples.

## 1. Introduction

Cryo-electron tomography (Cryo-ET) has emerged as a powerful 3D imaging tool with unprecedented quality in capturing structural and spatial organization information of macromolecules inside single cells. Analysis of macromolecules in a Cryo-ET image (i.e., a tomogram, usually of size 6,000 × 6,000 × 1,500 voxels) is done at subtomogram level. A subtomogram is a small 3D cubic sub-image of a tomogram that generally contains one macromolecule extracted from tomograms. Deep-learning-based classification has been successfully applied and achieved high accuracy on Cryo-ET subtomogram identification. Plenty of previous works have been devoted to separating structurally highly heterogeneous macromolecules captured by Cryo-ET data into structurally homogeneous subgroups (Bartesaghi et al., 2008; Scheres et al., 2009; Xu and Alber, 2011, 2012; Xu et al., 2012; Chen et al., 2014; Bharat et al., 2015; Che et al., 2018). Nevertheless, the main bottleneck for these deep learning methods is a lack of training data. Since various subtomogram datasets may be collected under different experimental conditions, directly applying the knowledge learned from one dataset to the other will result in a decrease in performance such as classification accuracy due to domain shift. Therefore, part of the dataset must be manually labeled in order to predict the rest of the data, which is a highly time consuming process. To automate this process and reduce domain shift, training the network on realistically generated subtomogram datasets becomes an ideal approach. Simulation can provide an unlimited number of training instances with pre-specified labels.

Conventional image simulation methods for Cryo-ET use atomic models in Protein DataBank (PDB) (Bernstein et al., 1977), using a specified resolution and voxel spacing together with low-pass data filtering. Gaussian-distributed noise and Modulation Transfer Function noise (MTF) are applied for the realistic electron optical effect to match a certain signal-to-noise ratio (SNR). Random rotation and translation operations are performed to synthesize more samples. Yet, simulating realistic data presents challenges due to high degree of structural complexity, irregular noise, and tomographic distortions. Neural networks trained on them result in poor testing performance when applied to experimental data. By inferring from real image data, machine learning methods potentially overcome common restrictions such as infeasible interactive use and substantial computational resources.

The recent explosion in the Generative Adversarial Networks (GANs) field have shown great success in tasks such as image synthesis, image-to-image translation (Yang et al., 2017; Schlemper et al., 2018; Seitzer et al., 2018; Wang et al., 2019, 2021; Guo et al., 2020; Yuan et al., 2020; Chen J. et al., 2021; Chen Y. et al., 2021; Jiang et al., 2021; Li et al., 2021; Lv et al., 2021a,b,c). Recent advances have used GANs to formulate biomedical image simulation as an image-to-image translation task and arouse a wide interest in biomedical area (Bi et al., 2017; Calimeri et al., 2017; Nie et al., 2017; Wolterink et al., 2017; Zhao et al., 2017; Liu et al., 2021a,b). In most cases, 3D images do not have paired data; as a result, learning from unpaired data becomes crucial. The cycle-consistent generative adversarial network (Zhu et al., 2017) successfully performed unpaired image-to-image translations, only requiring two unpaired datasets and is capable of preserving semantics. In the same spirit, we formulate a framework called CryoETGAN to simulate subtomograms indiscriminable from real data on given structures from density map which shows electron density occupancies and distribution of the particle (Kaur et al., 2021). We conduct experiments to demonstrate the effectiveness of our method qualitatively and quantitatively. The generated datasets can serve as training datasets for future subtomogram study.

We are the first to propose an image translation based simulation method for cryo-ET 3D images. Although image translation has been used to simulate cryo-EM 2D images (Gupta et al., 2020b, 2021; Miolane et al., 2020), they are not directly comparable to our method as 3D cryo-ET and 2D cryo-EM images capture different kinds of information. One prior work applying GANs in a related space is Gupta et al. (2020a), in which a GAN is trained to perform single-particle cryogenic electron microscopy (Cryo-EM) reconstruction given a large number of Cryo-EM images. We note this work differs in many aspects including the task and the nature of the data. First, Gupta et al. (2020a) trains a generative simulator using many Cryo-EM images of a specific particle, not a general image-to-image translation model. In addition, 2D single-particle cryogenic electron microscopy (Cryo-EM) images and 3D cryo-electron tomography (Cryo-ET) images are different media: single-particle Cryo-EM typically uses noisy images of many copies of a macromolecular structure, while Cryo-ET operates on a single cell sample (Marx, 2018). As noted in Marx (2018), Cryo-ET shines where it is not feasible to make “tens of thousands” of copies of a structure of interest, and has led to discoveries such as Basler et al. (2012). In essence, Gupta et al. (2020a) solves an important but distinct task in a related field.

Thus, our main contributions are as follows:

1. We propose the use of a GAN-based image translation method in order to augment the training datasets of Cryo-ET models using density maps.
2. We develop a GAN framework to robustly generate diverse Cryo-ET images from density maps. We propose several architectural modifications to incorporate priors on Cryo-ET data to stabilize training.
3. We demonstrate the effectiveness of these techniques on traditional metrics of generative model performance as well as downstream classification performance.

## 2. Materials and Methods

**Figure 1:**
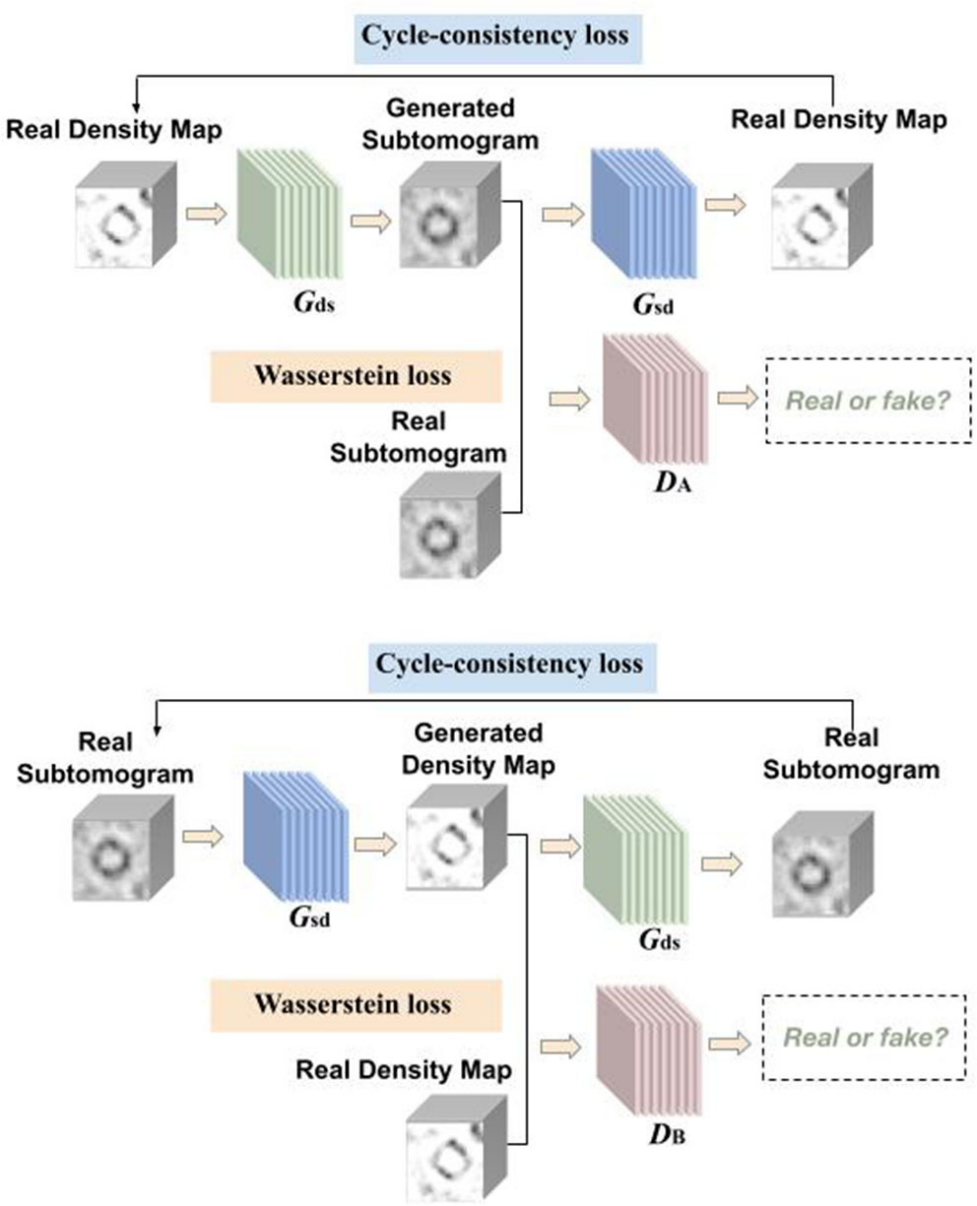
Overview of CryoETGAN: with adversarial loss, cycle-consistency loss, and Wasserstein loss, our method is capable of learning mapping between domain S and D with unpaired data.

Our proposed framework for Cryo-ET image synthesis: CryoETGAN is presented in Figure 1. In the following paragraphs, we will elaborate on CryoETGAN and its network architecture starting with preliminary details.

### 2.1. Formulation

We first introduce our notations. Macromolecular complexes and cellular components which can be extracted from tomograms of cells using template-free methods such as Difference of Gaussian, are densely packed in small 3D volume of cubic shape (3D analog of a 2D image patch). Those experimental subtomograms are represented as ${\left\{s_{i}\right\}}_{i=1}^{N}$ where *s*_*i*_ ∈ *S* (i.e., 3D gray scale images of size *n* × *n* × *n*).

Another domain we use contains density maps which are simulated from proteins using EMAN2 (Tang et al., 2007), which is a image processing package with a focus on single particle reconstruction. Those experimental density maps are denoted as ${\left\{d_{i}\right\}}_{i=1}^{N}$ where *d*_*i*_ ∈ *D*, our goal is to learn two mapping functions, *G*_*ds*_ : *D* → *S* and *G*_*sd*_ : *S* → *D*. The generators are guided by the discriminators to learn the mappings between the subtomograms and density maps in order to preserve the edges and details.

As shown in Figure 1, our CryoETGAN model has four main components: two generators *G*_*ds*_ and *G*_*sd*_ to capture the data distribution from two domains, two discriminators *D*_*A*_ and *D*_*B*_ that estimate the probability of the generated samples whether they are from the experimental datasets or generated ones. Discriminator *D*_*A*_ aims to distinguish between experimental subtomograms ${\left\{s_{i}\right\}}_{i=1}^{N}$ and generated ones ${\left\{G_{ds}\left(d_{i}\right)\right\}}_{i=1}^{N}$, and *D*_*B*_ aims to discriminate between experimental density map ${\left\{d_{i}\right\}}_{i=1}^{N}$ and generated ones ${\left\{G_{sd}\left(s_{i}\right)\right\}}_{i=1}^{N}$. Two generators are trained to produce realistic data to fool the adversarially trained discriminators *D*_*A*_ and *D*_*B*_. The training loss of CryoETGAN contains three types of terms: adversarial loss for matching the distribution of generated data to corresponding *D* or *S* domain; cycle-consistent loss to make sure the generated images in target domain can be generated back to the source domain and enable the mapping between these two domains; and Wasserstein loss to prevent mode collapse.

### 2.2. Adversarial Loss

The adversarial losses are applied to both mapping directions. Given a distribution *s* ~ *p*_data_, generators define the probability distribution as the distribution of the sample *G*_*ds*_(*d*) and *G*_*sd*_(*s*) For the generator *G*_*ds*_ : *D* → *S* and its discriminator *D*_*A*_, the objective is defined as:


(1) $$\begin{array}{l} {ℒ}_{\text{GAN}}\left(G_{ds},D_{A},D,S\right)=E_{s\sim p_{\text{data}}(s)}\left[\log D_{A}(s)\right]\\ \;+E_{d\sim p_{\text{data}}(d)}[\log\left(1-D_{A}\left(G_{ds}(d)\right)\right] \end{array}$$

In this setting, we train the generators *G*_*ds*_, *G*_*sd*_, and discriminators *D*_*A*_, *D*_*B*_ together. Without paired data, we conduct a min-max training between the generators and discriminators. Ideally the image *G*_*ds*_(*d*) generated by *G*_*ds*_ will be visually similar to images in *S* domain. Meanwhile the discriminators distinguish between generated images and real images. Similarly, the adversarial loss for the mapping function *G*_*sd*_ : *S* → *D* and its discriminator *D*_*B*_ is defined as below: $\underset{G_{sd}}{\min}\underset{D_{B}}{\max}L_{\text{GAN}}\left(G_{sd},D_{B},S,D\right)$


(2) $$\begin{array}{l} {ℒ}_{\text{GAN}}\left(G_{sd},D_{B},S,D\right)=E_{s\sim p_{\text{data}}(d)}\left[\log D_{B}(d)\right]\\ \;+E_{s\sim p_{\text{data}}(s)}[\log\left(1-D_{B}\left(G_{sd}(s)\right)\right] \end{array}$$

### 2.3. Cycle Consistency Loss

To further guarantee that the mapping function can map an input *d*_*i*_ to its ideal output *s*_*i*_, also from *s*_*i*_ to *d*_*i*_. Inspired by Zhu et al. (2017), we use cycle-consistent loss to enable the image translation cycle to force *d* back to the original image, i.e., *d* → *G*_*ds*_(*d*) → *G*_*sd*_(*G*_*ds*_(*d*)) ≈ *d*. Similarly, for each image *s* from domain *S*, *G*_*sd*_ and *D*_*d*_ should also make the reconstructed image *G*_*ds*_[*G*_*sd*_(*s*)] to be identical to input *s*. The cycle-consistent loss is written as:


(3) $$\begin{array}{l} {ℒ}_{\text{cyc }}\left(G_{ds},G_{\text{sd }}\right)=E_{x\sim p_{\text{data }}(d)}\left[{\left\|G_{\text{sd }}\left(G_{ds}(d)\right)-d\right\|}_{1}\right]\\ \;+E_{s\sim p_{\text{data }}(s)}\left[{\left\|G_{ds}\left(G_{sd}(s)\right)-s\right\|}_{1}\right]. \end{array}$$

### 2.4. Wasserstein Loss

During preliminary testing, expressions of density maps were frequently transferred to the same pose and to the same subtomogram expression. Moreover, the standard discriminator loss uses cross-entropy loss and suffers from vanishing gradients. Instead of the *Jensen-Shannon* divergence, Wasserstein GAN (Arjovsky et al., 2017) adopts the *Earth Mover* distance to measure the distance between real and generated samples:


(4) $$\begin{array}{l} W\left({\mathbb{P}}_{r},{\mathbb{P}}_{g}\right)=\underset{\gamma \in \Pi \left({\mathbb{P}}_{r},{\mathbb{P}}_{g}\right)}{\inf}{𝔼}_{\left(x,y\right)\sim \gamma }\left[\left||x-y|\right|\right]. \end{array}$$

Following the notation from Arjovsky et al. (2017) Π(ℙ_*r*_, ℙ_*g*_) represents for the set of all joint distributions. γ(*x, y*) represents for the transporting cost from *x* to *y* in order to transform the distributions ℙ_*r*_ to ℙ_*g*_. In practice, this is accomplished by replacing the discriminator with a critic and using the difference between the critic predictions on real and fake images as the critic's loss, and the negated version for the generator, and then enforcing a constraint on the discriminator to enforce 1-Lipschitz continuity. Inspired by Wasserstein GAN, we adopted the following improvements in order to deal with the model collapse problem in adversarial training and to achieve more stable results.

- Clip the weight ofs *D*.
- Use RMSProp instead of ADAM.
- Lower learning rate. The rate in the paper is α = 0.0005.

### 2.5. Mode Collapse

The scenario of mode collapse refers to the generator produces similar data every time and still able to successfully fool the discriminator. We pass random noise vectors to the generator in order to deal with mode collapse. To learn the distribution over subtomogram, the generator builds a mapping function from a distribution density map to subtomogram. Between convolutional layers and deconvolutional layers, we concatenate a noise vector to it so that it can generate different pattern according to the style. On the other side of the cycle translation, another generator builds a mapping function from subtomogram to density map.

### 2.6. Full Objective

Given the formulations of adversarial loss, cycle-consistent loss, and wasserstein loss above, our full objective is formulated as follows:


(5) $$\begin{array}{l} ℒ\left(G_{ds},G_{sd},D_{A},D_{B}\right)={ℒ}_{\text{GAN}}\left(G_{ds},D_{A},D,S\right)\\ \;+{ℒ}_{\text{GAN}}\left(G_{sd},D_{B},S,D\right)\\ \;+\lambda {ℒ}_{\text{cyc}}\left(G_{ds},G_{sd}\right), \end{array}$$

where λ adjusts the importance of the cycle-consistency objective.

Solving the min-max optimization problem has long been known for a challenging task. Previous work proposed careful designed network architectures and objective functions in order to achieve good performance—we adopt the spectral normalization layer proposed by Miyato et al. (2018) to normalize weights, regulating the scale of feature response values and stabilizing the training process.

### 2.7. Architecture

Following the CycleGAN paper notation (Zhu et al., 2017), the generator architecture is c7s1-d32, d64, d128, R128, R128, R128, R128, R128, R128, u64, u32, c7s1-u1. The output after downsampling is concatenated along the filter dimension with a one-channel Gaussian noise vector of the same shape, so the input to the u32 layer has 129 channels. Note dk denotes a k-filter 3 × 3 × 3 and stride-2 convolution followed by instance norm and ReLU, uk denotes the same with stride $\frac{1}{2}$ and fractional-strided-convolution, and Rk is a k-filter residual block. The last convolutional layer has tanh without InstanceNorm. The discriminator has an architecture of C64, C128, C256. Note Ck corresponds to a 4 × 4 × 4 convolution with stride 1 followed by InstanceNorm and a Leaky ReLU with slope of 0.2. Spectral Normalization is applied to each convolutional layer of the discriminator.

## 3. Results

### 3.1. Experimental Datasets

We tested our CryoETGAN on two experimental datasets *S*_*e*1_ and *S*_*e*2_. Dataset *S*_*e*1_ contains 1,600 subtomograms of size 40^3^ from four classes of macromolecules, the four classes are Proteasome (5MPA), Ribosome (5T2C), TRiC (4V94), and Membrane. Each class has 400 images. For the density maps, We simulated 3D noise free density maps using EMAN2 corresponding to the subtomogram classes. The proteins are from Protein Data Bank (Berman et al., 2000) which is a database for the three-dimensional structural data of large biological molecules, such as proteins and nucleic acids. Dataset *S*_*e*2_ contains 2,800 subtomograms from seven classes of macromolecules, which were extracted from Noble Single Particle Dataset collected by Noble et al. (2018), each class has 400 subtomograms from EMPIAR. Subtomograms were extracted and about 20 macromolecules were manually picked. The 20 subtomograms were averaged to generate the structural template. Structural template was aligned to all subtomograms extracted and produces cross-correlation scores. Each particle is consisted of 28^3^ voxels, and the size of each voxel is 0.94 nm. The SNR is 0.5 and missing wedge angle is 30°. For each tomogram in the original set, subtomograms of size 28^3^ were extracted using a Difference of Gaussian(DOG) particle picking process (Pei et al., 2016) with the parameters of *s*1 = 7.0 and *k* = 1.1. We applied a template search approach as described in Zeng et al. (2018) to select the top 1,000 subtomograms according to the cross-correlation scores. Four hundred subtomograms are manually selected for each class which contain macromolecule structures. In our experiments, we select 2,000 subtomograms for training and the remaining 800 for testing.

### 3.2. Visualization Results

**Figure 2:**
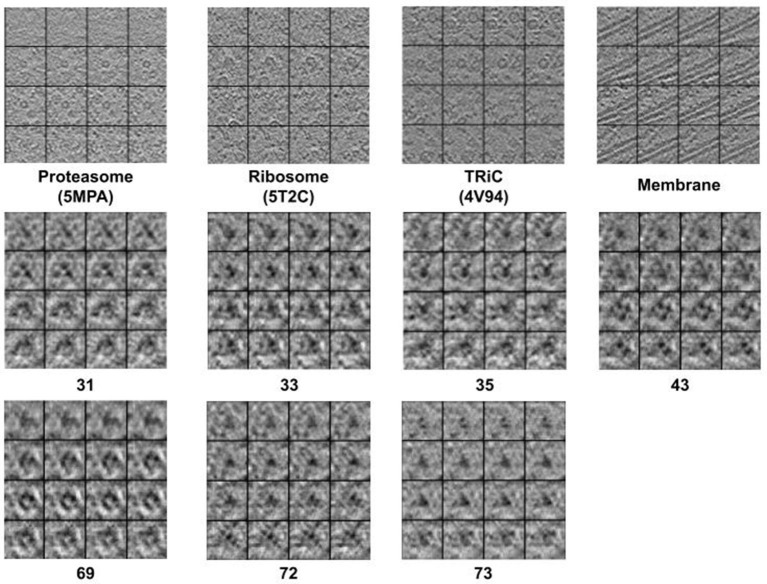
The 2D slide visualization of generated subtomograms (**Top**: *S*_*e*1_, **Middle and Bottom**: *S*_*e*2_). In general, we find CryoETGAN retrieval produces qualitatively similar subtomogram compared to the ground truth and is capable of producing various classes without mode collapse.

**Figure 3:**
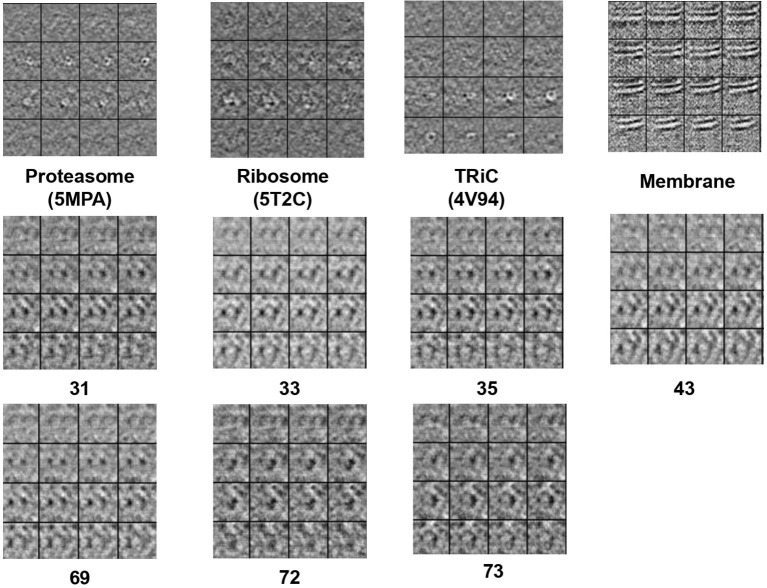
The 2D slide visualization of real subtomogram samples from every class (**Top**: *S*_*e*1_, **Middle and Bottom**: *S*_*e*2_), the sequence of those subtomograms is corresponding to the sequence in Table 1.

Figure 2 displays simulation results of applying CryoETGAN, and each section of the image represents for a slice of the generated subtomogram data. We can observe that the reconstructed images *G*_*sd*_[*G*_*ds*_(*d*)] end up matching closely to the input images *d* as shown in Figure 3.

**Table 1:** For domain *S*, we use two datasets for training *S*_*e*1_ and *S*_*e*2_ separately, which contains four classes subtomograms and seven classes subtomograms, with 400 images in each class.

***S*_*e*1_**	**EMPIAR ID**	**Macromolecular complex**
	5MPA	Proteasome
	5T2C	Ribosome
	4V94	TRiC
	NA	Membrane
***S*_*e*2_**	**EMPIAR ID**	**Macromolecular complex**
	10130&10131	Rabbit muscle aldolase
	10133	Glutamate dehydrogenase
	10135	DNAB helicase-helicase
	10143	T20S proteasome
	10169	Apoferritin
	10172	Hemagglutinin
	10173	Insulin-bound insulin receptor

### 3.3. Evaluation Metrics

We use several common GAN evaluation metrics (Borji, 2019) as the quality evaluation criteria for the Cryo-ET data generated in our experiments as shown in **Figures 5**, **6**.

#### 3.3.1. Inception Score (IS)

IS was originally proposed by Salimans et al. (2016) to quantitatively evaluate the quality of the generated images (shown in Equation 6). The intuition behind Inception Score is that a generator with high performance should generate samples with low entropy in the class distribution of a single generated data while producing high entropy in the classes across all generated samples. In our experiments, we adopted CB3D (Che et al., 2018) as our “Inception V3” to calculate an IS-equivalent for Cryo-ET.


(6) $$\begin{array}{l} \text{IS}=\exp\left({𝔼}_{x\sim p_{g}}D_{KL}\left(p\left(y|x\right)||p\left(y\right)\right)\right). \end{array}$$

#### 3.3.2. Frechet Inception Distance (FID)

FID has been widely used in measuring the similarity between real and generated images. Unlike IS, FID (Heusel et al., 2017) compares the distance between two multivariate Gaussian distributions as shown in (Equation 7)


(7) $$\begin{array}{l} \text{FID}=||\mu_{r}-\mu_{g}|{|}^{2}+\text{Tr}\left(\Sigma_{r}+\Sigma_{g}-2{\left(\Sigma_{r}\Sigma_{g}\right)}^{1/2}\right), \end{array}$$

s where $X_{r}\sim N\left(\mu_{r},\Sigma_{r}\right)$ and $X_{g}\sim N\left(\mu_{g},\Sigma_{g}\right)$ are the 4,096 dimensional activation inputs of the CB3D model's dense layer for real and generated data, respectively.

Single-value metrics such as IS and FID evaluate the generative model, yet they are not perfect for diagnostic purposes (Naeem et al., 2020). Fidelity and diversity attribute are usually considered as a trade-off in the design strategy of generative models, which represents for how realistic the inputs are and how well those generated data capture the variations in real data (Naeem et al., 2020). We use precision and recall proposed by Sajjadi et al. (2018) to measure these two characteristics, we use the same notations as in Naeem et al. (2020), B(X, r): the ball around the point x with radius r, NND_*k*_(*X*_*i*_): the distance to the kth-nearest neighbor. *X*_*i*_ are the real embedded samples and *Y*_*j*_ are the fake embedded samples.


(8) $$\begin{array}{l} \text{manifold}\left(X_{1},\cdots \;,X_{N}\right):=\overset{N}{\underset{i=1}{⋃}}B\left(X_{i},{\text{NND}}_{k}\left(X_{i}\right)\right). \end{array}$$

**Precision**:


(9) $$\begin{array}{l} \frac{1}{M}\overset{M}{\underset{j=1}{\sum }}1_{Y_{j}\in \text{manifold}\left(X_{1},\cdots \;,X_{N}\right)}. \end{array}$$

**Recall**:


(10) $$\begin{array}{l} \frac{1}{N}\overset{N}{\underset{i=1}{\sum }}1_{X_{i}\in \text{manifold}\left(Y_{1},\cdots \;,Y_{M}\right)}. \end{array}$$

#### 3.3.3. Density

Density and coverage are proposed by Naeem et al. (2020) as alternatives to precision and recall, respectively, to be more robust to outliers. Density emphasizes not only whether the samples generated are close to a real sample, but also how many spheres around real-samples contain the generated example. It counts how many real-sample neighborhood contains fake samples.


(11) $$\begin{array}{l} \frac{1}{kM}\overset{M}{\underset{j=1}{\sum }}\overset{N}{\underset{i=1}{\sum }}1_{Y_{j}\in B\left(X_{i},{\text{NND}}_{k}\left(X_{i}\right)\right)} \end{array}$$

#### 3.3.4. Coverage

Coverage is a metric evaluating recall in terms of the real manifold rather than the fake manifold. This penalizes sparse coverage of the real space, where generators may benefit in terms of the recall metric by simply having few examples in some part of the real space. It builds the nearest neighbor manifolds around the real samples instead of the fake samples due to more outliers.


(12) $$\begin{array}{l} \frac{1}{N}\overset{N}{\underset{i=1}{\sum }}1_{\exists \;j\text{s.t.}Y_{j}\in B\left(X_{i},{\text{NND}}_{k}\left(X_{i}\right)\right)} \end{array}$$

#### 3.3.5. Classification Accuracy

Deep Neural networks are able to capture global and local information from image data. Therefore, we use the state-of-the-art deep learning-based classification model for Cryo-ET data: CB3D (Che et al., 2018) to objectively quantify the generated subtomogram generated from density map data. We consider this as a way to interpret the generative ability of our model.

Compared to the traditional method (Bernstein et al., 1977) which has the testing classification accuracy 19.7% on a well-trained CB3D for *S*_*e*1_ and 28.9% for *S*_*e*2_, our method outperforms the traditional method by achieving the classification accuracy of 76.4 and 67.3%.

We believe that the fact that the coverage result is much better than the recall result is a consequence of a few factors: first, the relatively small size of the real dataset means that the original recall metric will penalize the model for generating anything except exactly the correct test set examples. Using the real manifold, as in coverage, rather than the fake manifold, as in recall, is more forgiving. Since these metrics were not developed with an emphasis on small real datasets and the evaluation of precision and recall of generative models is an ongoing topic of research, there may be a better metric to be proposed, but this is outside the scope of our article. The evaluation results are shown in Table 2.

**Table 2:** Evaluation results via six different metrics.

**Datasets**	**SSIM**	**Precision**	**Recall**	**Density**	**Coverage**	**Classification acc (%)**
*S*_*e*1_	0.3071	1.0	0.0	320.0	1.0	76.4
*S*_*e*2_	0.7192	0.3493	0.0678	2.21628	0.5532	67.3

*Ideally, one would have a high density as well as a high coverage. We believe these metrics alongside classification performance are the most relevant indicators for this model, as one density map may correspond to numerous subtomograms*.

### 3.4. Uncertainty Estimation

Uncertainty estimation is a common approach to check the generative model's performance, we build on Gal and Ghahramani (2016) and combine their contributions in order to get an uncertainty map using Monte Carlo dropout as an implicit representation of the underlying subnetworks.

**Figure 4:**
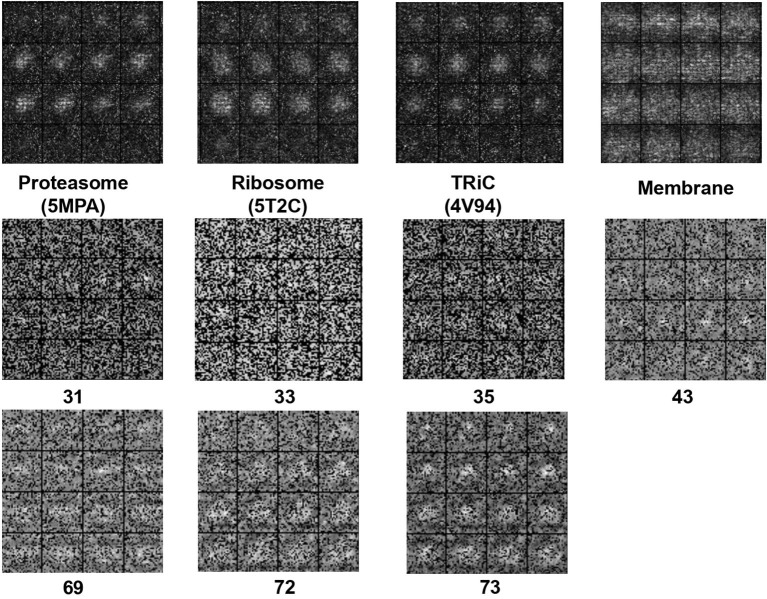
The 2D slide visualization of uncertainty map for (**Top**: *S*_*e*1_, **Middle and Bottom**: *S*_*e*2_).

The detailed description of our uncertainty estimation method is: we apply dropout in the generator, sample 20 times using the same density map, calculate the standard deviation per pixel, and then we can overlay them to have an uncertainty map over the pixel wise of the model per given input for visualization. Then we compare the result of using Dropout and not using dropout. In this way we will be able to measure the generator uncertainty from pixel level. We show the uncertainty maps in Figure 4.

### 3.5. Ablation Study

#### 3.5.1. Analysis of Noise Standard Deviation

**Figure 5:**
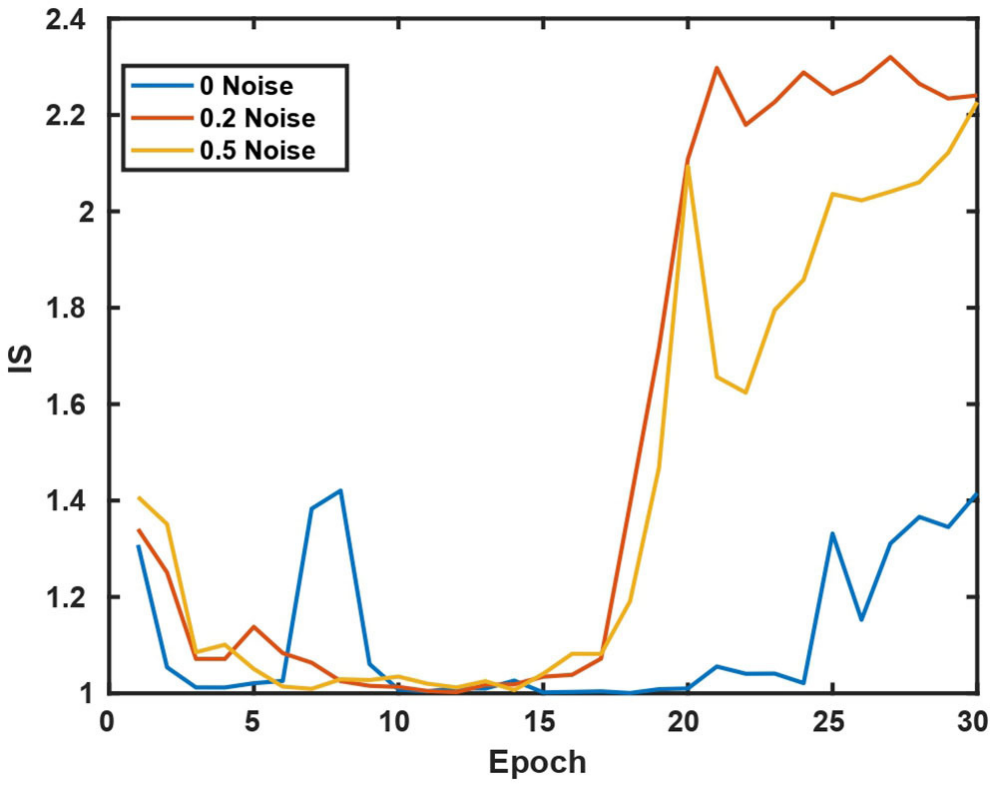
Inception Score w.r.t various standard deviations of noise.

**Figure 6:**
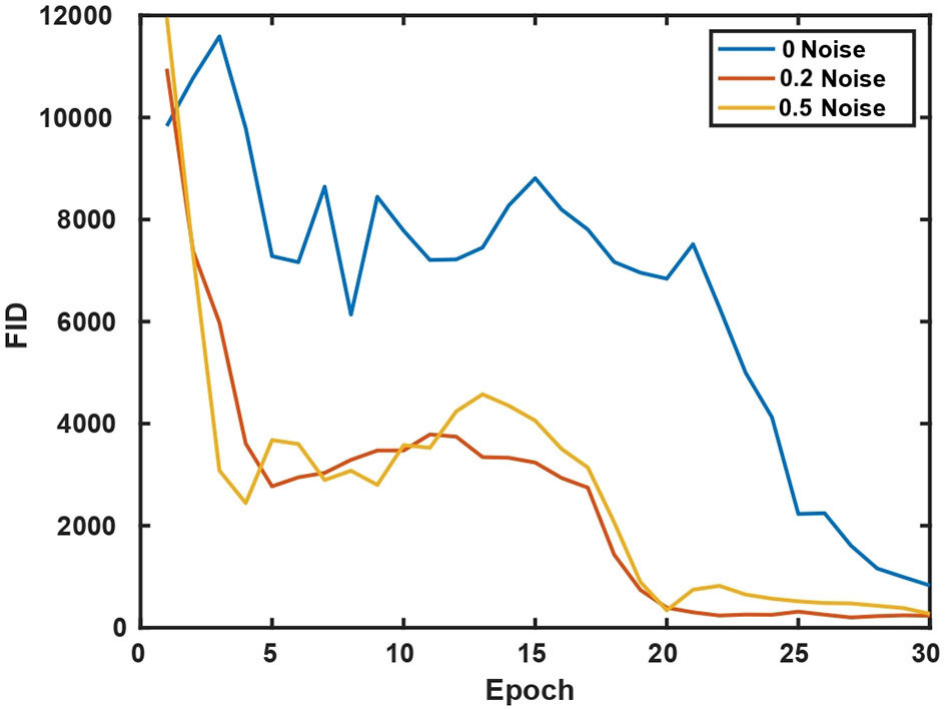
Frechet Inception Distance w.r.t various standard deviations of noise.

In Table 3, we compare CryoETGAN's performance under various standard deviations of noise during training. The performance of our CryoETGAN substantially improved when we applied zero-mean Gaussian noise to the density maps in the experiment relative to training without noise. From Figures 5, 6, we can see improvements in Inception Score and faster convergence in Frechet Inception Distance.

**Table 3:** Ablation study to demonstrate the performance impact of applying zero-mean Gaussian noise applied on density maps w.r.t. Frechet Inception Distance and Inception Score.

**GAN setup**	**Evaluation metrics**	
	**Frechet Inception Distance**	**Inception Score**
CryoETGAN without gaussian noise	828.18	1.42
CryoETGAN + 0.2 × gaussian noise	201.37	2.32
CryoETGAN + 0.5 × gaussian noise	273.01	2.22

#### 3.5.2. Analysis of Model and Loss Design

We further evaluated the presence of the Wasserstein loss and the Spectral normalization. The results are shown below. Here we evaluated on *S*_*e*1_ four classes dataset. We find that without the Wasserstein loss there is clear indication of mode collapse, and without the spectral norm a significant penalty on downstream performance. The ablation study results are shown in Table 4.

**Table 4:** Ablation study to demonstrate the performance impact of using Wasserstein loss and Spectral normalization.

**Wass**.	**Spec**.	**SSIM**	**Precision**	**Recall**	**Density**	**Coverage**	**Classification**
**loss**	**norm**						**acc. (%)**
✓	✓	0.3071	1.0	0.0	320.0	1.0	76.4
	✓	0.2006	0.0	0.0	0.0	0.0	26.9
✓		0.0413	1.0	0.0	320.0	1.0	57.1

*The results show that the wasserstein loss and the spectral normalization significantly improved the performance*.

## 4. Conclusion

We proposed a machine learning based method: CryoETGAN to synthesize Cryo-ET images and therefore to enable the realistic simulation of protein density maps consistent with the Cryo-ET data. Our generated images performed competitively when trained for classification and this approach potentially increases the available training data for further new Cryo-ET based algorithms which depends on large data collection. This new data provides a way to investigate new methods for object detection, segmentation, domain adaptation tasks, etc. Our approach can also be extended to support other multimodal nanoparticles image synthesis in fluorescence/soft X-ray/tomography of nucleoplasmic reticulum and apoptosis in mammalian cells, which serves as a way to study images and resolve tasks limited by insufficient available data.

## Data Availability Statement

The original contributions presented in the study are included in the article/supplementary material, further inquiries can be directed to the corresponding author/s.

## Author Contributions

MX conceived the study. XW and XZ proposed CryoETGAN. XW designed and implemented the methods and ran analysis. CL and HW evaluated the methods and did the ablation studies. XW, CL, and HW analyzed the results. XZ processed the data. XW wrote the article with suggestions from MX, XZ, H-WD, and JZ. All authors contributed to the article and approved the submitted version.

## Funding

This work was supported in part by U.S. NIH grants R01GM134020 and P41GM103712, NSF grants DBI-1949629 and IIS-2007595, and Mark Foundation For Cancer Research 19-044-ASP. We thank the computational resources support from AMD COVID-19 HPC Fund. XZ was supported in part by a fellowship from CMU CMLH. JZ was supported in part by U.S. NIH grant K01MH123896.

## Conflict of Interest

The authors declare that the research was conducted in the absence of any commercial or financial relationships that could be construed as a potential conflict of interest.

## Publisher's Note

All claims expressed in this article are solely those of the authors and do not necessarily represent those of their affiliated organizations, or those of the publisher, the editors and the reviewers. Any product that may be evaluated in this article, or claim that may be made by its manufacturer, is not guaranteed or endorsed by the publisher.
